# Chain length of dietary fatty acids determines gastrointestinal motility and visceromotor function in mice in a fatty acid binding protein 4-dependent manner

**DOI:** 10.1007/s00394-019-02094-2

**Published:** 2019-09-27

**Authors:** Paula Mosińska, Adrian Szczepaniak, Tatiana Wojciechowicz, Marek Skrzypski, Krzysztof Nowak, Jakub Fichna

**Affiliations:** 1 Department of Biochemistry, Faculty of Medicine, Medical University of Lodz, Mazowiecka 6/8, 92-215 Lodz, Poland; 2 Department of Animal Physiology and Biochemistry, Poznań University of Life Sciences, Poznan, Poland

**Keywords:** Medium-chain fatty acids, Long-chain fatty acids, Coconut oil, Fatty acid binding protein 4, Gastrointestinal motility, Irritable bowel syndrome

## Abstract

**Purpose:**

We hypothesize that different types of dietary fatty acids (FAs) affect gastrointestinal (GI) motility and visceromotor function and that this effect can be regulated by the fatty acid binding protein 4 (FABP4).

**Methods:**

Mice were fed for 60 days with standard diet (STD), STD with 7% (by weight) coconut oil, rich in medium-chain FAs (MCFAs) (COCO), or with 7% evening primrose oil, rich in long-chain FAs (LCFAs) (EPO). In each group, half of the mice received FABP4 inhibitor, BMS309403 (1 mg/kg; i.p.) twice a week. Body weight (BW) and food intake were measured; well-established tests were performed to characterize the changes in GI motility and visceral pain. White adipose tissue and colonic samples were collected for cell culturing and molecular studies.

**Results:**

COCO significantly increased GI transit, but not colonic motility. COCO and EPO delayed the onset of diarrhea, but none affected the effect of loperamide. EPO reduced BW and increased the visceromotor response (VMR) to colorectal distension (CRD). COCO and EPO reduced differentiation of preadipocytes. Treatment with BMS309403: (1) reversed the effects induced by COCO in physiological conditions and in mouse models of diarrhea; (2) prevented the effects of EPO on BW, VMR to CRD and castor oil-induced diarrhea; (3) affected proliferation of preadipocytes; (4) changed the expression of *Fabp4* in colonic and adipocyte samples from COCO and EPO.

**Conclusion:**

Modifying dietary intake of MCFAs and LCFAs may be used to control GI motility or visceral pain and thus modulate the symptoms of functional GI disorders. The effect is dependent on the expression of FABP4.

**Electronic supplementary material:**

The online version of this article (10.1007/s00394-019-02094-2) contains supplementary material, which is available to authorized users.

## Introduction

Functional gastrointestinal disorders (FGIDs) are a heterogeneous group of chronic conditions that considerably reduce the patient’s quality of living, interfering with their social life, education and working ability. The most common FGID noted in the population worldwide is irritable bowel syndrome (IBS) [1], whose prevalence in Europe and North America ranges from 10 to 20% [2–4]. According to the symptom-based classification system (the Rome IV criteria), IBS is a chronic, relapsing bowel disorder associated with the following symptoms: stool irregularities, visceral hypersensitivity, altered mucosal function with concomitant psychiatric and somatic comorbidities [5]. Depending on patterns of symptoms, IBS is classified as IBS-C (constipation-predominant), IBS-D (diarrhea-predominant) or IBS-A (alternating, including both diarrhea and constipation); however, the symptoms may vary and oscillate not only between subtypes but also within the same patient over time. Due to the complex nature of IBS and only a partially understood pathogenesis, this condition still poses an immense challenge in the twenty-first century for both general practitioners and clinicians.

Ingestion of food components has long been linked with IBS symptoms. At least two-thirds of IBS patients reported food as an important trigger for worsening their condition [6] and thus, many of them avoid certain dietary components. Due to the fact that food is a complex milieu of several chemicals, it is hard to pinpoint one particular group or type of nutrient responsible for the onset or worsening of symptoms. In the recent years, clinicians have suggested that the reduction of fiber intake or the consumption of fermentable oligo-, di-, monosaccharides and polyols (FODMAP) may minimise the occurrence of symptoms [7–10].

There are little data addressing the lipid turnover in IBS [2, 11–14]. Park et al. [15] showed in rats that dietary fats attenuated the motile function of the entire GI tract and delayed gastroduodenal transit. Available data evaluating dietary fat intake between IBS patients and control individuals are inconsistent, yet it undoubtedly indicates a disturbed level of several fatty acids (FAs) in their serum. For example, Clarke et al. [16] reported increased concentrations of n-3 polyunsaturated fatty acids (PUFAs) in the serum of IBS patients, compared to the control group. In contrast, Solakivi et al. [17] showed that the proportions of arachidonic acid and the FAs belonging to the family of PUFAs were significantly decreased in subjects with IBS, compared to controls. Of note, the same study highlighted that IBS group had higher concentrations of unsaturated and monounsaturated FAs, and lower concentrations of n-3 PUFA, compared to controls. There were also attempts to assess the effects of administering lipids to IBS subjects. A study by Caldarella et al. [12] showed that intraduodenal lipid infusion of a low-calorie fatty meal decreased the rectal sensory threshold in comparable fashion in both IBS-C and IBS-D. Another study documented changes in the pattern of viscerosomatic pain referral in IBS patients and increased their sensitivity for gas sensation and perception of urge [13]. Although the enhancement of intestinal sensitivity occurred independent of the type of IBS, gender or psychological factors, the mechanism behind it remains uncertain.

Several studies have suggested the involvement of selected proteins secreted by the white adipose tissue (WAT) in the pathophysiology of IBS [18–20]. Fatty acid binding protein 4 (FABP4) is predominantly present in adipocytes, where it is able to bind to hydrophobic ligands, including eicosanoids, saturated and unsaturated FAs, and participate in their metabolism and excretion from the body. It has been recently shown that mouse and human gut epithelial Paneth cells express FABP4 and are simultaneously the main source of its presence in the intestines [21].

Our previous studies showed that the mRNA expression of FABP4 was significantly decreased in colonic samples of IBS-C patients, when compared to the control group indicating the active contribution of the protein in the course of IBS [22]. We also demonstrated that the acute administration of the FABP4 inhibitor, BMS309403, increased motility in the mouse lower GI tract in physiological conditions and in pharmacologically delayed GI transit.

Since the function of the lower digestive tract can be affected by ingestion of FAs, we aimed to determine the effects of dietary FAs on the lower GI motility and visceral pain. We also verified whether the FABP4 pathway is in charge of the effects observed by the implementation of dietary modifications. Finally, to better understand the role of FABP4 in the lipid turnover, we characterized the effects of treatments on adipocyte differentiation and proliferation.

## Materials and methods

### Animals and study design

Experimentally naive male BALB/c mice, weighing 22–24 g were obtained from the vivarium at the University of Lodz, Poland. The animals were housed under controlled laboratory conditions (22–23 °C, relative humidity: 45–55%, 12:12 h light/dark cycle, lights on at 6:00 a.m.) in sawdust-lined plastic cages. Tap water was available ad libitum. To minimize circadian influence, all experiments were performed between 7:00 h and 16:00 h after at least 7 days of acclimatization. After acclimatization, the weight-matched animals were randomly assigned to three groups fed, respectively, with standard laboratory diet (STD) containing 7% fat by weight, diet supplemented with 7% coconut oil, rich in medium-chain FAs (MCFAs) (COCO), or diet supplemented with 7% evening primrose oil, rich in long-chain FAs (LCFAs) (EPO). COCO group resulted in a diet rich mostly in lauric and myristic FAs, whereas EPO enrichment results in a diet with high content of linoleic acid. Animals were fed for 60 days with free access to water. All diets were formulated to meet the nutritional requirements of growing mice and manufactured by the external company specialized in animal food supply (ZooLab, Poland) (Table 1) [23]. Body weight was measured twice a week; the food intake was monitored every day in the morning between 7 and 8 a.m. Experimental groups comprised 5–10 animals to provide statistically relevant data.

**Table 1:** Composition of diets that will be included in the study

Ingredients (g/kg diet)	STD	STD supplemented with 7% coconut oil (COCO)	STD supplemented with 7% evening primrose oil (EPO)
Corn starch	397.486	397.486	397.486
Casein	200.0	200.0	200.0
Maltodextrin	132.00	132.00	132.00
Soybean oil	70.00	0.00	0.00
Coconut oil/evening primrose oil	0.0	70.00	70.00
Fiber	50.00	50.00	50.00
^a^AIN93G mineral mix	35.00	35.00	35.00
^a^AIN93G vitamin mix	10.00	10.00	10.00
l-Cystine	3.00	3.00	3.00
Choline bitartrate	2.50	2.50	2.50
Tert-butylohydrochinon	0.014	0.014	0.014
	1000.0	1000.0	1000.0

Full ingredient list for the diets in this study, formulated by ZooLab, Poland

^a^AIN93 refers to rodent diet 93 of the American Institute of Nutrition [23]

Two parallel studies were performed:

1. Mice (*n* = 72) were randomly allocated to three diet groups (STD, COCO or EPO, 24 mice/group) and fed for 60 days. Animals were subsequently used to assess the GI motility in physiological conditions i.e. colon bead expulsion test and fecal pellet output (FPO) and under pathophysiological conditions, i.e. mouse models mimicking constipation and diarrhea. Twelve animals in each dietary intervention received the intraperitoneal (i.p.) injection of the FABP4 inhibitor, BMS309403 (1 mg/kg) twice a week [22]. The experiments were performed in two-day intervals.
2. Mice (*n* = 60) were randomly allocated to three diet groups (STD, COCO or EPO, 20 mice/group) and fed for 60 days. Ten animals in each group were additionally administered with BMS309403 (1 mg/kg, i.p.) twice a week. Subsequently, the colorectal distention (CRD) was performed to determine the visceromotor response (VMR) to pain.

**Fig. 1:**
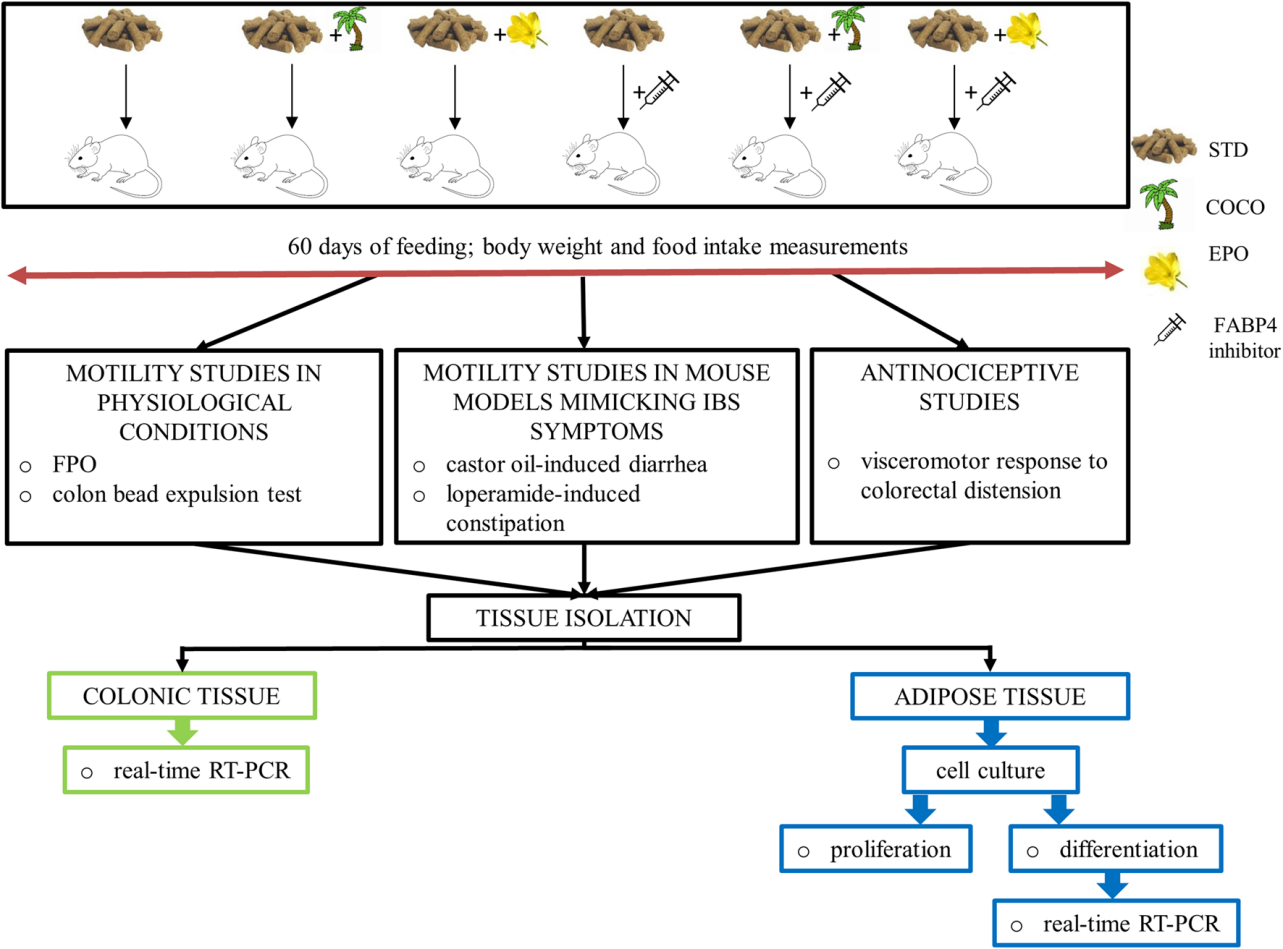
Schematic representation of the experiments described in the paper

A schematic representation of the experiments included in the study is shown in Fig. 1.

The experimental protocols followed the European Communities Council Directive of 22 September 2010 (2010/63/EU), were in accordance with Polish legislation on animal experimentations, and were approved by the Local Ethics Committee at the Medical University of Lodz (#63/LB77/2017). All efforts were made to minimize animal suffering and to reduce the number of animals used.

BMS309403 and loperamide were dissolved in 5% dimethyl sulfoxide (DMSO) and diluted in 0.9% saline to desired concentrations as selected in preliminary studies. 5% DMSO dissolved in 0.9% saline was used as vehicle. BMS309403 was injected intraperitoneally (i.p.) at the dose of 1 mg kg^−1^ twice a week for 60 days. For mouse models mimicking IBS, loperamide or castor oil was administered acutely at the dose of 3 mg kg^−1^ (i.p., 100 µL/mouse) and 0.2 mL/mouse per os (p.o.), respectively, 15 min and 10 min before the respective experiment. Control animals received vehicle alone (100 µL/mouse, i.p.). The vehicles in the used concentrations had no effect on the observed parameters in mice. Mice used in VMR to CRD were anesthetized by the i.p. injection of ketamine/xylazine solution 1 mL and 0.5 mL, respectively, diluted in 8.5 mL of 0.9% sodium chloride.

## Materials

All drugs and reagents were purchased from Sigma-Aldrich (Poznań, Poland), unless stated otherwise. BMS309403, a FABP4 inhibitor, and loperamide were purchased from Tocris (Bristol, UK). Xylazine and ketamine were acquired from Biovet (Puławy, Poland). Isoflurane was obtained from Baxter Healthcare Corp. (IL, USA). PBS, in the form of ready-to-use tablets, was purchased from Polgen (Lodz, Poland). TRIsure was obtained from Bioline (London, UK). The PCR TaqMan Gene Expression Assay probes used for the quantification of FABP4 mRNA expression were purchased from Life Technologies (CA, USA). Dulbecco’s modified Eagle’s medium Nutrient Mixture F-12 (DMEM/F12) for cell culture was obtained from Thermo Fisher Scientific, Inc. (Waltham, USA).

### Gastrointestinal motility studies

#### Physiological conditions

To measure whole GI transit, the fecal pellet output (FPO) was assessed in non-fasted animals [24] after 60 days of feeding. Mice were placed individually into clean transparent cages with no access to water or chow. Sixty minutes later, the number of excreted fecal pellets was counted as a measure of GI tract motility.

To measure colonic motility, distal colonic bead expulsion test was performed after 60 days of feeding following an overnight fasting (for 16 h) with free access to drinking water, as described previously [24]. On the day of the experiment, a prewarmed (37 °C) glass bead (2 mm) was inserted 2 cm into the distal colon using a silicone pusher under light isoflurane anesthesia. After bead insertion, mice were separated into transparent, individual cages and the time to bead expulsion was measured up to 15 min after administration of BMS309403 or control vehicle [25].

#### Pathophysiological conditions

Constipation was induced by the i.p. administration of peripherally restricted mu-opioid receptor agonist, loperamide, 15 min before the FPO test [26].

Diarrhea was induced by oral administration of castor oil as previously described by Fichna et al. [27]. Immediately after the castor oil administration, the animals were placed into individual wire-bottomed cages. The symptoms of diarrhea were evaluated depending on time between the administration of castor oil and diarrhea-related symptoms, i.e., excretion of liquid feces [28].

The effects of chronic administration (twice a week for 60 days) of BMS309403 on the GI motility were compared with the mean transit observed in the control (STD + BMS) group.

### Cell studies

#### Isolation of mouse white preadipocytes

Primary adipocytes were isolated from epididymal fat pads from male BalbC mice, according to the methods by Skrzypski et al. [29] and Chatteryee et al. [30] with few modifications. The collected adipose tissue was purified from blood vessels and washed three times in sterile Krebs-Ringer buffer [118 mM NaCl, 1.3 mM CaCl_2_, 4.8 mM KCl, 1.2 mM MgSO_4_, 1.2 mM KH_2_PO_4_, 24.8 mM NaHCO_3_, 10 mM HEPES 4-(2-hydroxyethyl)-1-pipera-zineethanesulfonic acid] supplemented with 3% bovine serum albumin, antibiotics (100 U/mL penicillin and 0.1 mg/mL streptomycin) and 5 mM glucose. The washed tissue was minced with scissors. The adipose tissues isolated from each mouse belonging to the same experimental group were pooled together. Thereafter, pooled tissues were digested in Krebs-Ringer HEPES buffer (KRBH) containing collagenase type II (3 mg/mL), BSA, streptomycin and glucose (5 mmol/l) for 45 min at 37 °C in a shaking water bath. After the incubation, growth medium containing 10% fetal bovine serum, penicillin and streptomycin was added to the flask. After digestion, lysate was centrifuged at 450×*g* for 10 min at room temperature (RT). To lyse the erythrocytes, the Red Blood Cell Lysing buffer (Sigma-Aldrich) was added to pellet. To discard the remaining undigested tissue debris, cells were filtered through a nylon mesh (100 µm). Next, cells were filtered through a 40-μm mesh. Filtered cells were centrifuged at 450×*g* to separate floating adipocytes from stromal-vascular cell pellets. The suspension containing mature adipocytes was discarded. The cell pellet was resuspended in DMEM/F12 containing antibiotics and 10% fetal bovine serum and counted using a Fuchs-Rosenthal counter chamber with 0.4% trypan blue (cell viability > 95%). Cells were seeded in multi-well plates and incubated under humified atmosphere (5% CO_2_ and 95% air).

#### Cell proliferation

Cell proliferation was assessed using a cell proliferation ELISA BrdU colorimetric kit (Sigma-Aldrich). Isolated mouse preadipocytes were seeded in 96-well plates and cultured for 24 h. Cell culture was conducted at 37 °C in a humidified atmosphere (95% air with 5% CO_2_). Cells were deprived of serum for 24 h to synchronize the cell cycle. Subsequently, 10 µL of 10 µM BrdU solution was added and incubated with cells for 2.5 h. The incorporation of BrdU into DNA was measured colorimetrically using Cell Proliferation ELISA BrdU colorimetric kit (Roche Diagnostic, Penzberg, Germany) according to manufacturer protocol ending with colorimetric measurement at 450 nm.

#### Cell differentiation

Differentiation of preadipocytes into adipocytes was induced by DMDM/F12 medium containing adipogenesis-promoting agents (2 nM triiodothyronine, 167 nM insulin and 30 nM dexamethasone), according to the standard protocol [31]. The effect of differentiation on adipocytes was determined on days 1, 3 and 6. Successful differentiation was assessed by morphology of cells and compared with standard marker of *Fabp4* [32]. Cells were harvested at the days 1, 3 and 6 and stored at − 80 °C in TRIsure reagent (Roche Diagnostics, Basel, Switzerland) for RNA extraction.

### Visceromotor response (VMR) to colorectal distension (CRD)

The method used to evoke the model of visceral pain was performed as previously described [27]. Mice were anesthetized with ketamine/xylazine solution and underwent the surgery: two electrode wires were implanted into the abdominal oblique muscles. The ends of wires were pulled under the skin toward the incision on the neck and externalized. Incisions on neck and abdomen were tightly sutured; the ends of electrodes were mounted to the mouse’s skin using stitches and tapes. All surgical procedures were carried out in line with the antiseptic policy according to the approved animal protocol guidelines. Animals were allowed to rest for 2 days in individual cages. The measurement of VMR to the pressure stimulus was conducted on day 3. Each mouse was placed in a restraint to allow easy access to the tips of wires. Fogarty’s catheter (Thru-lumen embolectomy catheter, Fogarty, Edwards Lifescience, Warsaw, Poland) was used to induce the pressure in the large intestine. Lubricated balloon end of catheter was inserted 5 mm proximal to the anus and taped to the tail. A reference electrode was attached to the mouse tail and all electrodes were placed in the appropriate plugs. VMR to four values of pressure was measured: 15 mmHg, 30 mmHg, 45 mmHg and 60 mmHg acquired by incrementally insufflating the balloon with distilled water (0.2 mL, 0.4 mL, 0.6 mL 0.8 mL, accordingly) for 10 s with 5-min intervals. The 10-s stimulus was applied only once per pressure value. Electromyograms were acquired using Bio Amp (ADInstruments, Poznan, Poland), connected to PowerLab and a personal computer with Lab Chart 7 software. Electromyogram amplitudes in millivolts (mV) were collected over the period of 10 s for the baseline (before the stimulus) and for the response to stimulus. Data are presented as the difference between the VMR induced by the distension and the baseline, expressed as the area under the curve.

### Quantitative analysis of FABP4

#### Mouse colonic tissue preparation

The segments from the distal colon of each animal were resected. Fecal contents, as well as connective tissue residues were gently removed and rinsed with phosphate-buffered saline (PBS). Colon samples were transferred into new tubes and stored at − 80 °C until protein analysis.

#### Quantification of FABP4 mRNA expression in mouse tissue and harvested cells

For the quantification of mRNA expression, we applied the real-time fluorescence detection PCR method with FAM dye-labeled TaqMan probes (Applied, Biosystems, USA). The colonic mouse RNA was isolated according to the manufacturer’s protocol using Total RNA Mini kit (A&A Biotechnology, Poland). Briefly, colonic tissue samples were homogenized in TRIsure reagent (Bioline, London, UK) using an ultrasound homogenizer (Bandelin Sonoplus HD3100, Berlin, Germany), whereas harvested differentiated cells (pellet cells) were lysed with TRIsure and the cell lysate was further homogenized by passing cells through an insulin syringe three times. Subsequently, all samples were centrifuged (11,000×*g* for 10 min at 4 °C), placed onto silica columns and purified. Total RNA was eluted using diethyl pyrocarbonate treated water. The purity and quantity of the isolated RNA were measured using dedicated spectrophotometer (iMarkTM, BioRad Laboratories). The sample was characterized with A260 nm/A280 nm ratio, which was in the range of 1.79–2.01. Total RNA was then transcribed into cDNA with Maxima First Strand cDNA synthesis kit (Fermentas, Canada) with the following three-step incubation: 25 °C for 10 min, 50 °C for 15 min and 85 °C for 5 min. Quantitative analysis was performed using fluorescently labeled TaqMan probes for mouse *Fabp4* and for mouse hypoxanthine–guanine phosphoribosyltransferase 1 (*Hprt1*) as endogenous control (Life Technologies, Carlsband, CA, USA) on Mastercycler S realplex 4 apparatus (Eppendorf, Germany) and TaqMan Gene Expression Master Mix (Life Technologies, Carlsbad, CA, USA) in accordance with the manufacturer’s protocol. The catalog numbers for the probes used are as follows: FABP4—Mm00445878_m1, HPRT1—Mm01545399_m1. The real-time reaction mixture was prepared in a total volume of 20 µL and consisted of 1 µL of cDNA, 10 µL of TaqMan Gene Expression Master Mix, 8 µL of RNA-free water and 1 µL of FAM dye-labeled TaqMan probes. All experiments were performed in triplicate. The threshold cycle (Ct) values for studied genes were normalized to Ct values obtained for a housekeeping gene, *Hprt1*. The relative expression levels were normalized to *Hprt1* and calculated as 2^[− (Ct_FABP4_ − Ct_HPRT1_)] × 1000 [33].

### Statistical analysis

Statistical analysis and curve-fitting were performed using Prism 5.0 (GraphPad Software Inc., La Jolla, CA, USA). The data are expressed as mean ± SEM. One-way ANOVA followed by the Student–Newman–Keuls post hoc test was used for analysis of multiple treatment means. *p* values < 0.05 were considered statistically significant. The data and statistical analysis comply with the recommendations on experimental design and analysis in pharmacology [34].

## Results

### Long-term dietary supplementation with evening primrose oil significantly changed the body weight of mice

**Fig. 2:**
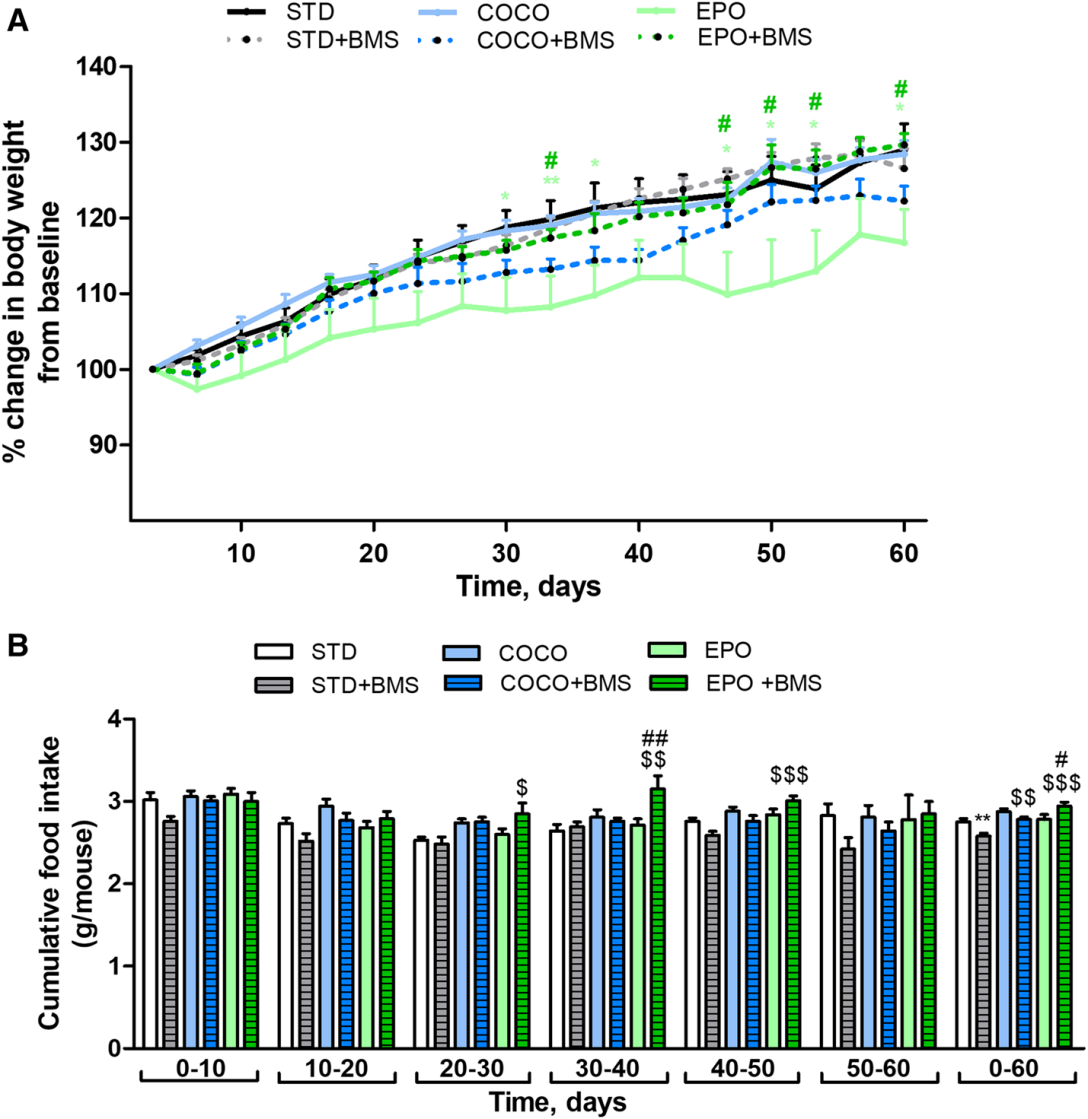
Representative graphs for body weight changes (**a**) and food intake (**b**) in mice fed with standard diet (STD), standard diet supplemented with coconut oil (COCO) and standard diet supplemented with evening primrose oil (EPO) with and without treatment with BMS309403 (1 mg/kg; i.p.). Data represent mean ± SEM of *n* = 10 mice per group. *p < 0.05 and **p < 0.01 vs. STD; ^$^*p* < 0.05, ^$$^*p* < 0.01 and ^$$$^*p* < 0.001 vs. STD with BMS309403; ^#^*p* < 0.05 and ^##^*p* < 0.01 vs. respective dietary intervention without BMS309403 (one-way ANOVA followed by post hoc Newman–Keuls multiple comparison test)

As shown in Fig. 2a, the animals fed with EPO presented with the lowest body weight gain after 60 days of feeding. The change was noticeable from the first day of feeding and maintained during the whole dietary intervention period. Significant differences were observed between EPO and STD groups as well as between EPO and the COCO groups, particularly between the 30th and 36th, 46th and 53rd and the 60th days of treatment (*p* < 0.05). In contrast, supplementation with coconut oil (COCO group) did not change the body weight of animals and showed comparable values with STD group.

To determine the possible mechanism responsible for the observed changes in body weight of animals from COCO and EPO groups, half of the animals were additionally injected with BMS309403, the FABP4 inhibitor at the dose of 1 mg/kg (i.p.). BMS309403, administered twice a week for 60 days, slightly reduced the body weight in COCO + BMS; although, this was not significant. No changes in the body weight were observed in the EPO + BMS group, when compared to STD + BMS group. However, a significant increase in the body weight in EPO + BMS group vs. EPO group was noted in 32nd, 46th, 50th, 53rd and 60th days of dietary intervention (*p* < 0.05).

### Long-term dietary supplementation with coconut oil or evening primrose oil did not affect food intake in mice

We did not observe any significant differences in the cumulative food intake between STD, COCO and EPO groups exposed to 60 days of feeding (Fig. 2b). In contrast, animals from the STD + BMS group ingested significantly less than those from STD (*p* < 0.01), whereas the animals fed with EPO + BMS—significantly more than those on EPO diet (*p* < 0.05). No difference between the COCO + BMS and COCO groups was noted (Fig. 2b).

Mice from EPO + BMS group consumed more of their meal compared to BMS309403-treated animals being on the STD diet (STD + BMS group). A significant increase was first observed from the 30th day of feeding (*p* < 0.05) and continued until the 50th day (*p* < 0.001). The treatment with BMS309403 also increased the average cumulative food intake (0–60 days) in COCO + BMS and EPO + BMS groups (*p* < 0.01 and *p* < 0.001 vs. STD + BMS group, respectively).

### Diet supplemented with the coconut oil significantly increased mouse GI transit and BMS309403 administration reversed this effect

**Fig. 3:**
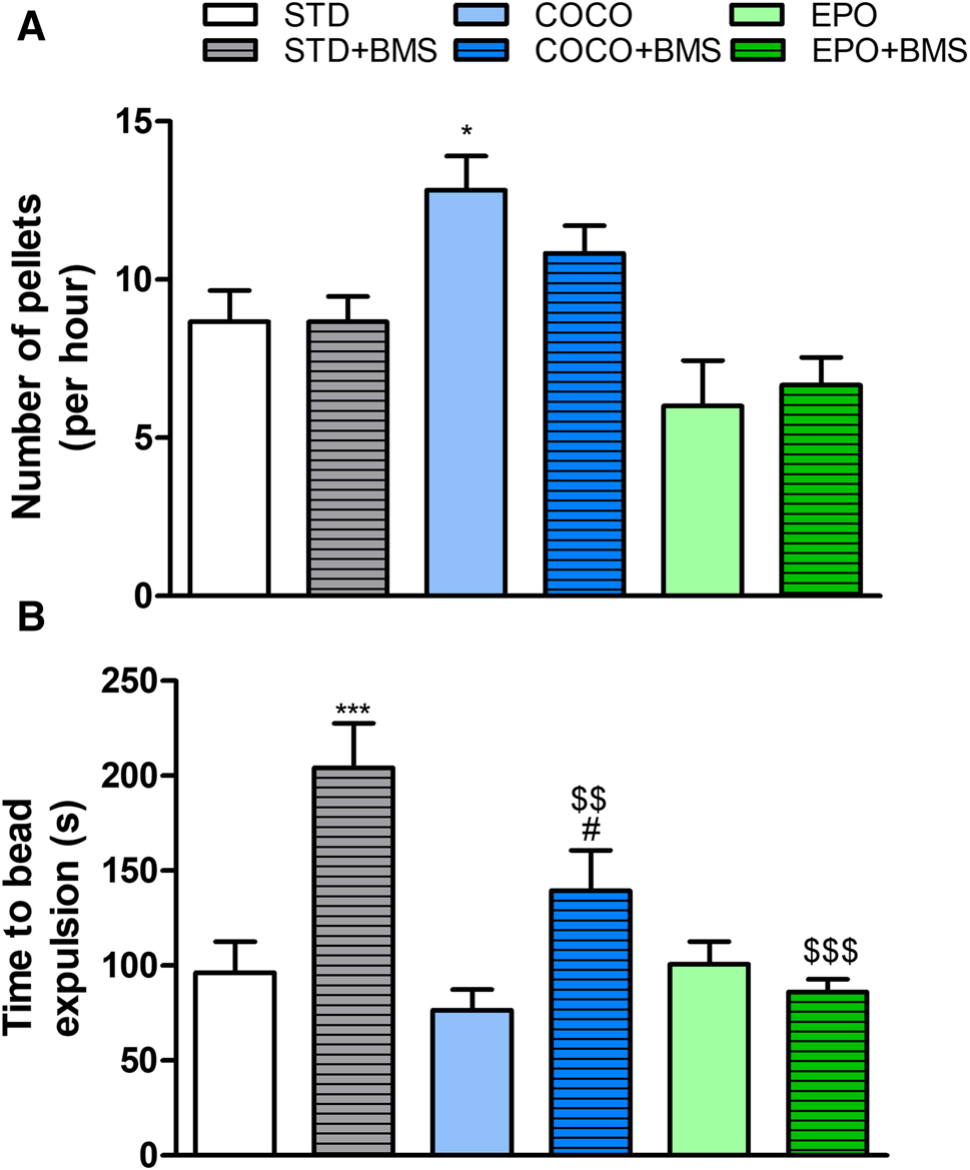
The effect of different dietary interventions (STD, COCO and EPO) with or without the administration of BMS309403 (1 mg/kg, i.p.) on fecal pellet output (**a**) and colonic transit in mice (**b**). Data represent mean ± SEM of *n* = 5–6 mice per group. *p < 0.05, ***p < 0.001 vs. STD; ^$$^*p* < 0.01 and ^$$$^*p* < 0.001, vs. STD with BMS309403; ^#^*p* < 0.05 vs. respective dietary intervention without BMS309403 (one-way ANOVA followed by post hoc Newman–Keuls multiple comparison test)

FPO was used to verify the effects of supplementation with COCO and EPO on mouse GI transit. In the COCO group, the GI transit was accelerated in mice under physiological conditions, resulting in a significant increase in the number of pellets excreted over 60 min (*p* < 0.05 vs. animals fed with standard diet, STD) (Fig. 3a). In contrast, the defecation pattern of mice fed with EPO diet did not change when compared to animals on STD diet.

Chronic administration of the selective FABP4 inhibitor, BMS309403, at the dose of 1 mg/kg (i.p.) delayed the GI transit in COCO + BMS group. The intestinal motor activity in the STD + BMS and EPO + BMS groups was similar to the respective dietary modifications without the BMS309403 intervention (animals belonging to EPO and STD, respectively) (Fig. 3a).

### Dietary supplementation with coconut oil or evening primrose oil had no effect on the mouse colonic transit in physiological conditions

To characterize the effects of each diet (STD, COCO and EPO) on colonic motor function, we performed the colonic bead expulsion test. None of the diets produced significant changes in the colonic motility (Fig. 3b).

The i.p. injection of BMS309403 in STD + BMS and COCO + BMS groups prolonged the time to bead expulsion, reflecting a significant inhibitory effect on propulsive motility in the lower GI tract (*p* < 0.001 vs. STD and *p* < 0.05 vs. COCO, respectively) (Fig. 3b). Colonic motility pattern in the EPO + BMS group did not change after the injection of FABP4 inhibitor compared to the EPO group.

### Chronic administration of BMS309403 to mice fed with coconut oil significantly reversed loperamide-induced hypomotility

Effects of dietary supplementations and the FABP4 inhibitor, BMS309403, on the GI motility were also assessed in the mouse model mimicking constipation.

**Fig. 4:**
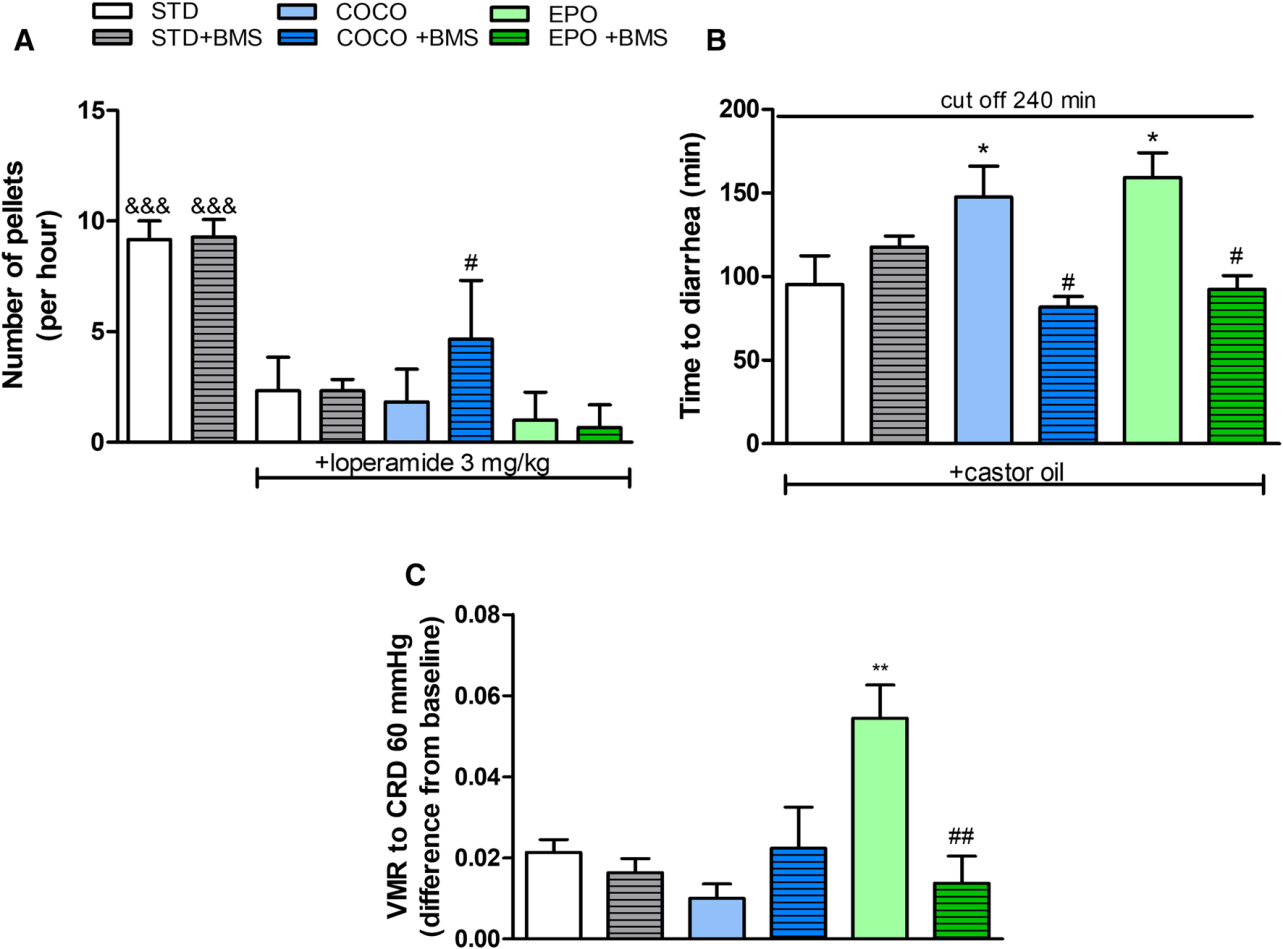
The effect of different dietary interventions (STD, COCO and EPO) with or without the administration of BMS309403 (1 mg/kg, i.p.) on mouse GI motility and visceromotor response to colorectal distention (**c**). The GI motility was assessed in the mouse model of loperamide–induced hypomotility (3 mg mg/kg, i.p.) (**a**) and in castor oil-induced diarrhea (200 µl, p.o.) (B). Data represent mean ± SEM of *n* = 5–10 mice per group. *p < 0.05 and **p < 0.01 vs. STD; ^&&&^*p* < 0.001 vs. STD + loperamide (3 mg/kg, i.p.); ^#^*p* < 0.05 and ^##^*p* < 0.01 vs. respective dietary intervention without BMS309403 (one-way ANOVA followed by post hoc Newman–Keuls multiple comparison test)

We verified whether the effect induced by loperamide could be reversed by either dietary modification or administration of the BMS309403. We observed that in all experimental groups (STD, STD + BMS, COCO, COCO + BMS, EPO and EPO + BMS) loperamide, an opioid anti-diarrheal agent, significantly inhibited the GI motility (*p* < 0.001 vs. STD or STD + BMS groups, not injected with loperamide) (Fig. 4a). Dietary modifications did not change the effect induced by the loperamide itself; however, chronic injection of BMS309403 at the dose of 1 mg/kg (i.p.) to mice fed with coconut oil (COCO + BMS) accelerated colonic transit over two times when compared to COCO group (*p* < 0.05) (4.667 ± 1.08 and 1.833 ± 0.600, respectively).

### Chronic administration of BMS309403 to mice fed with either coconut oil or evening primrose oil significantly accelerated the onset of diarrhea

We investigated the impact of chronic dietary supplementation with oils and the effect of the inhibition of FABP4 in the mouse model of diarrhea. The diarrhea was induced by the oral administration of castor oil, which causes accumulation of water and electrolytes in the intestine. In our study, both the supplementation with coconut oil (COCO group) and evening primrose oil (EPO group) delayed the onset of diarrhea, when compared to mice on a standard diet (STD group) (Fig. 4b).

Mice belonging to the COCO and EPO groups, which were additionally injected with BMS309403, had significantly reduced time to appearance of liquid feces when compared with respective dietary intervention without BMS309403 (*p* < 0.05; 81.80 ± 6.34 for COCO + BMS vs 147.5 ± 18.54 for COCO, and 92.40 ± 8.23 for EPO + BMS vs 158.8 ± 14.96 for EPO). The administration of BMS309403 to mice fed with STD diet did not affect time to occurrence of diarrhea.

### Supplementation with EPO increased VMR to CRD and BMS309403 administration reversed this effect

To assess changes in visceral sensitivity between groups supplemented with oils and groups treated with BMS309403, we measured VMR to CRD. The pressure values ranging from 15 to 45 mmHg did not affect the pain perception of animals. The maximum pressure used in the study (60 mmHg) caused a significant increase in response to pain induced by balloon distention in EPO group, measured as the area under the curve (*p* < 0.01 vs. STD) (Figure S-1); the effect was reversed after the administration of BMS309403 (*p* < 0.01 vs. EPO + BMS). Figure 4c shows the effects of 60 mmHg on VMR to CRD in all experimental groups.

### Dietary supplementation does not affect the expression of *Fabp4* in the mouse colon

**Fig. 5:**
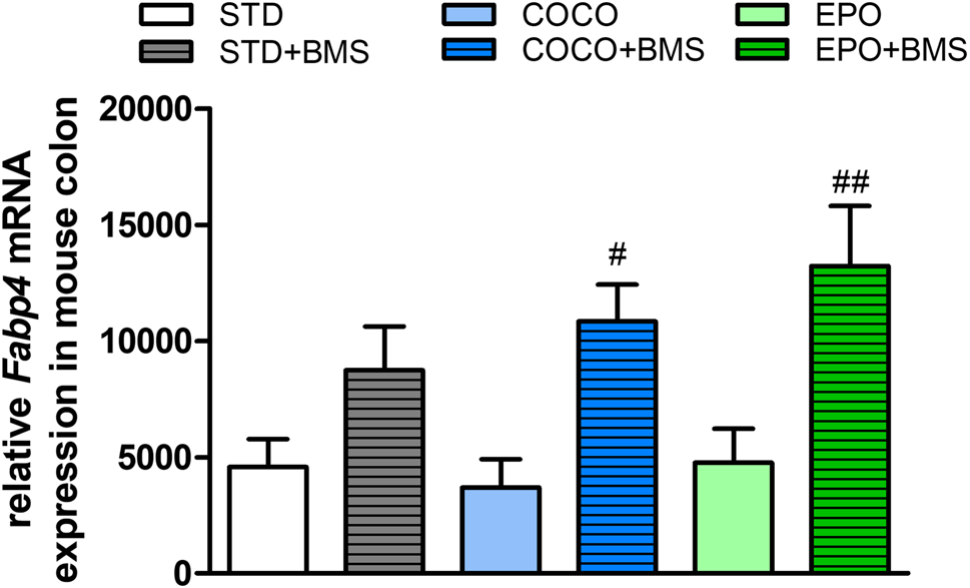
The influence of different dietary interventions (STD, COCO and EPO) with or without the injection of BMS309403 (1 mg/kg, i.p.) on relative mRNA expression of FABP4 in mouse colonic tissue. Data represent mean ± SEM of *n* = 5–6 mice per group. ^#^*p* < 0.05 and ^##^*p* < 0.01 vs. respective dietary intervention without BMS309403 (one-way ANOVA followed by post hoc Newman–Keuls multiple comparison test)

To further characterize the influence of dietary supplementation and the effects of the pharmacological blockage of FABP4, we measured mRNA expression of *Fabp4* in mouse colonic tissues. Fatty acid supplementation had no effect on the expression of *Fabp4* in the colon (Fig. 5). Dietary supplementation with simultaneous administration of BMS309403 increased the mRNA expression of *Fabp4* in COCO + BMS and EPO + BMS groups (*p* < 0.05 vs. COCO and *p* < 0.01 vs. EPO, respectively).

### Only diet with evening primrose oil suppressed the proliferation of preadipocytes

**Fig. 6:**
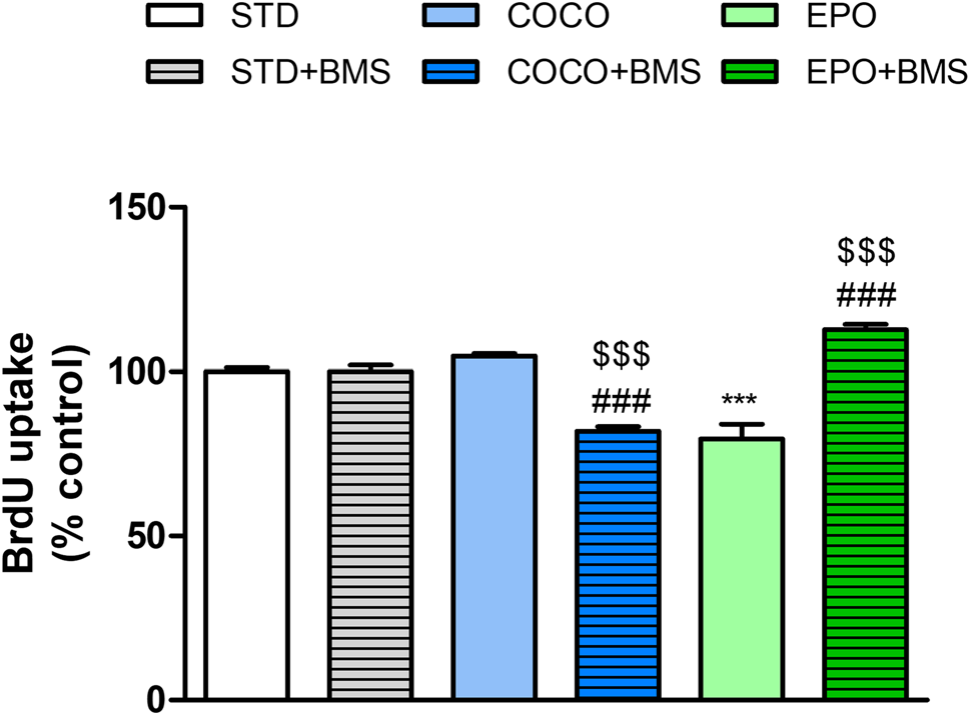
The effects of 60 days of feeding with either a standard chow (STD), a STD supplemented with coconut oil (COCO) or STD supplemented with the evening primrose oil (EPO), without or with the administration of BMS309403, on preadipocyte proliferation. Data are expressed as mean ± SEM for *n* = 24. ***p < 0.001, vs. cells isolated from the STD-fed mice; ^$$$^*p* < 0.001, vs. STD with BMS309403; ^###^*p* < 0.001, vs. respective dietary intervention without BMS309403 (one-way ANOVA followed by post hoc Newman–Keuls multiple comparison test)

Subsequently, we examined whether preadipocyte proliferation can be affected by diet or chronic treatment with BMS309403. As shown in the Fig. 6, the proliferation of cells isolated from the EPO-treated animals was significantly attenuated by 20% (*p* < 0.001, vs. STD group); COCO diet failed to produce any significant changes.

In contrast, the administration of FABP4 inhibitor BMS309403 significantly suppressed the proliferation of COCO + BMS-derived preadipocytes by 19% vs. STD + BMS, and by 23% vs. COCO. BMS309403 counteracted the anti-proliferative effect seen in EPO, leading to increased survivability of the EPO + BMS cells for about 12% (*p* < 0.001, vs. STD + BMS) and 32% vs. EPO (Fig. 6).

### The expression of *Fabp4* in preadipocytes differed between COCO and EPO groups as well as between groups treated with BMS309403

**Fig. 7:**
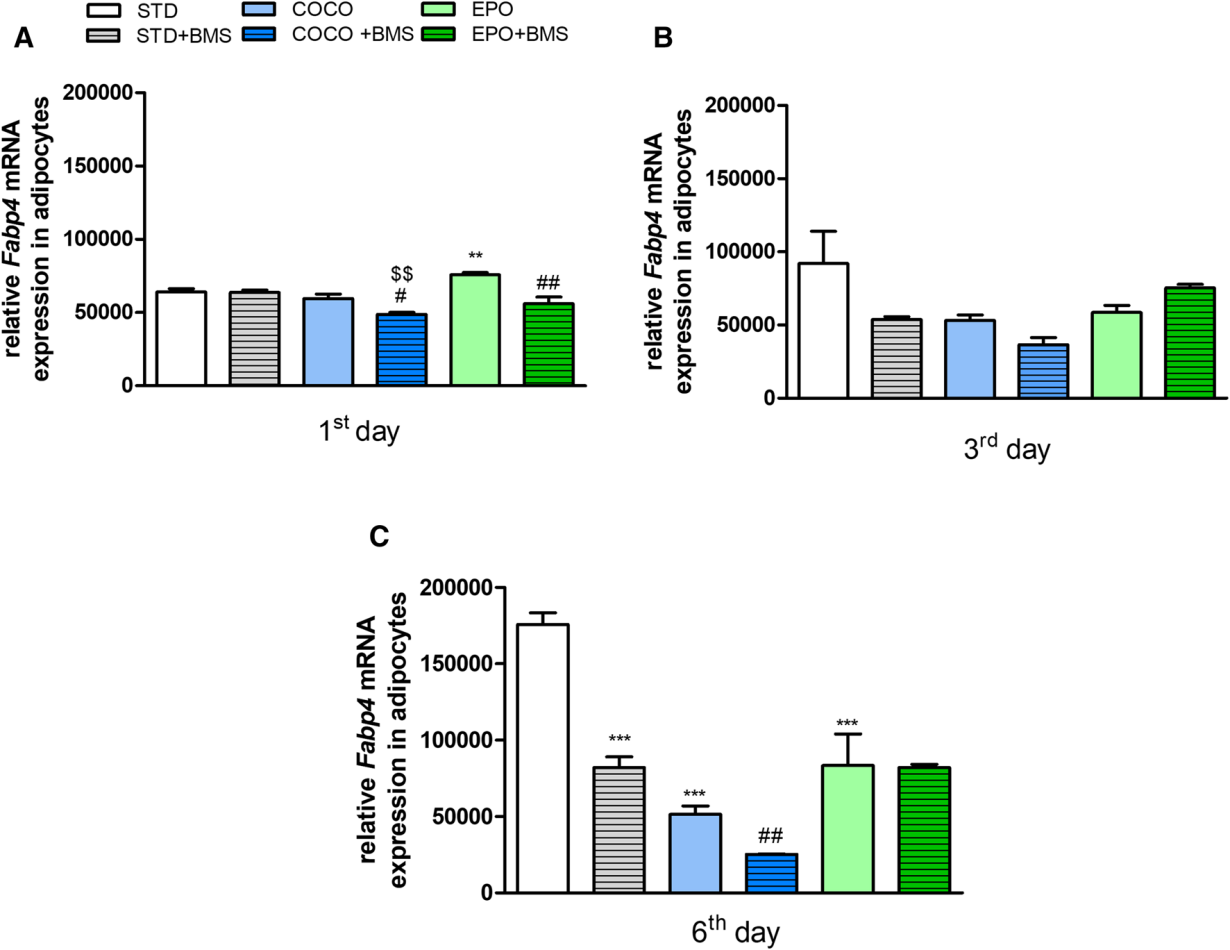
The relative mRNA expression of *Fabp4* in preadipocytes isolated from animals fed with either standard chow (STD), standard chow supplemented with coconut oil (COCO) or evening primrose oil (EPO), without and with the administration of BMS309403 (1 mg/kg, i.p.). The cells were harvested on the 1st (**a**), 3rd (**b**) and 6th (**c**) days following the induction of differentiation. Data are expressed as mean ± SEM **p < 0.01, ***p < 0.001, vs. cells isolated from the STD-fed mice; ^$$^*p* < 0.01, vs. STD with BMS309403; ^#^*p* < 0.05, ^##^*p* < 0.01, vs. respective dietary intervention without BMS309403 (one-way ANOVA followed by post hoc Newman–Keuls multiple comparison test)

We measured the expression of *Fabp4* in preadipocytes isolated from mice fed with STD, COCO and EPO, and in mice which additionally received the treatment with the BMS309403 (Fig. 7).

Feeding with COCO had no effect on the *Fabp4* expression in preadipocytes, whereas the supplementation with EPO significantly increased its level on the 1st day following the onset of differentiation (*p* < 0.01 vs. cells from STD-fed mice) (Fig. 7a). On the 3rd day, the expression of *Fabp4* decreased in both the COCO and EPO groups, reaching the statistical significance on day 6 following the induction of differentiation (*p* < 0.001 vs. STD-derived cells).

BMS309403 injected twice a week during the whole period of feeding changed the expression of *Fabp4* in preadipocytes isolated from the COCO and EPO groups (Fig. 7). On the 1st day of differentiation, a significant decrease in the relative mRNA expression of *Fabp4* was observed in preadipocytes derived from the COCO + BMS group, when compared to the STD + BMS (*p* < 0.01) and COCO groups (*p* < 0.01 and *p* < 0.05, respectively). The mRNA expression of *Fabp4* cells isolated from the EPO + BMS group was significantly reduced only when compared to the EPO group (*p* < 0.01). On the 3rd day, the expression of *Fabp4* in the STD + BMS and COCO + BMS groups decreased; however, these results were not significant. EPO + BMS did not show any differences vs. STD + BMS or EPO groups. On the 6th day, the mRNA expression of *Fabp4* in the STD + BMS and COCO + BMS groups remained decreased and showed statistical difference vs. STD (*p* < 0.001) and COCO (*p* < 0.01) groups, respectively. No changes in the expression of *Fabp4* in the EPO + BMS group were observed.

## Discussion

There is a guideline consensus that diet and lifestyle advice should be the first-line approach in dietary treatment of IBS. Regular eating pattern, healthy eating habits (i.e., limiting the intake of potential dietary triggers, such as FODMAPs, spicy foods, caffeine or alcohol), and regular physical activity are typical lifestyle modifications recommended by specialists [35–37]. A vast proportion of patients with IBS relate their symptoms to foods rich in fat [38, 39]. Despite the limited evidence on the association between the intake of fats, and in particular of FAs, and the occurrence of IBS symptoms, elimination or the avoidance of food rich in fat is one of the approaches considered to improve patient’s well-being and their quality of life [40, 41]. Since laboratory-based studies are scarce and the clinical studies limited, we aimed at evaluating changes in the GI motility and visceral pain of animals exposed to different dietary interventions, and determine whether the observed effects can be regulated through the inhibition of the FABP4-dependent pathway.

Our results demonstrated that mice fed with diet rich in MCFAs (COCO group) showed typical body weight gain throughout the experiment. This corroborated previous outcome that weight gain in vivo depends on the FA composition of the diet, and that diet rich in MCFAs is non-obesogenic [42–44]. This may come from the fact that MCFAs are readily absorbed from the intestines directly into the portal vein, transported to the liver where they undergo beta-oxidation via carnitine-independent mechanism, and thus increase diet-induced thermogenesis. Our outcomes are in line with other observations [45–47].

In contrast, we noticed a substantial drop in the body weight in the EPO group, i.e., animals supplemented with LCFAs, with no effect on food intake. These effects of EPO are mainly attributed to the content of gamma-linolenic and linoleic acids (LA), which are precursors of omega-3 FAs, such as docosahexaenoic and eicosapentaenoic acids. Conjugated LA (CLA), a product of symbiotic fermentative bacteria which transform LA into its conjugated form, is considered as a bioactive compound, which exhibits anti-obesity effects by influencing lipid metabolism, i.e., reduces fat accumulation in adipose tissue and increases hepatic lipogenesis. Accordingly, CLA reduced body fat accumulation by increasing energy expenditure and endurance capacity within one week of feeding, and sustained it for more than 6 weeks without affecting the food intake [48]. Since CLAs are a family of isomers of LAs that could be found in EPO, it is also possible that the reduction in body weight seen in our study could be related to more intense locomotor activity of animals, which resulted in increased lipid metabolism. Albeit, as we did not evaluate energy expenditure or the exercise performance of animals throughout the study, we can only speculate about their possible contribution to the observed effect. Likewise, other studies also reported a substantial increase in the voluntary movement between 4  and 20 weeks of feeding with CLA, and decrease or no change in the body weight [49–52].

Concurrently, throughout the period of feeding, the cumulative food intake in both COCO and EPO groups was similar to STD, which provides additional evidence that supplementation with MCFAs, derived from coconut oil, or LCFAs, found in evening primrose oil, do not influence the appetite of animals. These findings are in accordance with other studies [52–54].

FABP4 was first identified in mature adipocytes and macrophages but recently its high expression was also detected in intestinal epithelial cells and in colonic mouse samples [21, 22]. Although FABP4 exhibits higher affinity towards saturated FAs, it also binds to unsaturated FAs and retinoic acid. The protein not only facilitates FA transport and metabolism, but also acts as signaling molecule that distributes or sequesters FAs to control signaling pathways and gene expression. Various studies showed that FABP4-deficient mice gained more weight upon induction of dietary obesity [55, 56]. Moreover, given the fact that FABP4 exerts higher affinity towards LCFAs than MCFAs, the increase in body weight was much more visible in the EPO + BMS group than in the COCO + BMS group at the end of the study. Besides the differences observed between COCO + BMS or EPO + BMS and respective dietary intervention without the injection of BMS309403, a remarkable change in cumulative food intake has also been noted between the STD + BMS and EP + BMS groups (between 30 and 60 days of feeding). It additionally indicates that the amount of food consumed by animals depended not only on the presence of BMS309403, but also on the content of FAs in a diet. The more PUFAs in a diet, the higher the amount of food consumed by animals.

Dietary FA supplementation influences GI motility in vivo; the effect it generates is dependent on the chain length and the type of FAs consumed. So far, both MCFAs and LCFAs have been evaluated mainly in regards of their effects on upper gut motility; few studies have focused on their effects on the lower GI motility. We hypothesized that dietary supplementation with coconut oil, which results in higher content of lauric acid (12 carbon atoms), can increase the bowel motility and that the effect would be greater than after the supplementation with evening primrose oil, in which the major component is the linoleic acid (18 carbon atoms). As expected, animals treated with MCFAs (COCO group) had significantly increased GI motility, which was evaluated based on the number of pellets excreted within a specified period of time. In contrast, animals supplemented with LCFAs (EPO group) did not show any differences when compared to STD group. Moreover, none of the diets affected colonic motility, which may suggest that the effect of FAs on motility depends on the location of FAs in the GI tract, i.e., the same type of FAs can exert different, even opposite effects whether it concerns the upper part of the digestive tract or the large intestine. The radiographic studies in rats showed that the supplementation with 3.5% EPO resulted in a slightly faster propel of the barium from the stomach to the colon when compared to animals supplemented with 3.5% soybean oil [57]. Although the matter content reached the colon faster, the EPO diet induced the strongest (but still not statistically significant) inhibitory effect on propulsive colonic motility. In humans, indirect comparison of results from different studies showed that LCFAs with ≥ 12 carbon atoms slow gastric emptying and affect antropyloroduodenal motility [58] more than MCFAs with ≤ 12 carbon atoms [59]. The intragastric administration of lauric acid, a saturated FA, to humans caused relaxation of the fundus and reduced the amplitude of antral contractions. The effect was more intense than after the infusion of capric acid (10 carbon atoms), which markedly stimulated duodenal pressure waves and had no effects on antral pressure waves. Other studies evaluated the effects of intraduodenal infusion of FAs to humans. The studies revealed that intraduodenal infusion of lauric acid stimulated pyloric motility and increased the number and amplitude of phasic and basal pyloric pressure waves more than the infusion of oleic acid (a monounsaturated FA with 18 carbon atoms). As concluded by the authors, the greater the load of lauric acid, the greater the stimulation of basal pyloric pressure and decrease in antral and duodenal pressure [59]. Taking into consideration that the animals in our study consumed similar amount of chow, and that the percentage of FAs in each diet was equal (7%), the observed changes in GI motility could only be attributed to the mechanism of action of a particular type of FA included in a diet. Nevertheless, it would be of particular importance for the future studies to 1) measure the amount of FAs that reach the intestine; 2) evaluate whether the increase in GI motility occurred as a result of greater (or certain) area of the small intestine that contacted the FA and therefore modulates their absorptive capacity; and 3) highlight the correlation between the chain length of FA and their absorption in the intestines.

In our study, the type of FAs in a diet neither significantly affected the colonic motility nor the mRNA expression of *Fabp4* in the colon. However, the administration of BMS309403 remarkably delayed the colonic motility of mice treated with STD + BMS and COCO + BMS, when compared to their respective dietary intervention without BMS309403, and slightly increased the motility in the EPO + BMS group vs. EPO. Moreover, the mRNA expression of *Fabp4* in BMS309403-treated animals varied between groups. The changes were dependent on the type of FAs present in the diet. Our results suggest that the longer the chain length of FAs, the higher the mRNA expression of *Fabp4* in the colon and the greater increase in colonic motility of animals treated with BMS309403. Since LCFAs are considered as more potent ligands for FABP4 than MCFAs, the inhibition of this protein may result in higher accumulation of LCFAs in colonic lumen.

Constipation and diarrhea are major ailments of the GI tract, which are common amongst patients with IBS. To characterize the effects of coconut oil and evening primrose oil supplementation, we employed experimental mouse models of pharmacologically induced constipation and used the laxative properties of castor oil to induce diarrhea. The treatment with loperamide caused a significant reduction of the intestinal transit, when compared to non-constipated animals, and this effect was maintained regardless of the applied interventions. Loperamide exerts its activity through the µ opioid receptors, which are expressed in the intestines. Since none of the interventions increased the effect of loperamide, it can be presumed that the expression of these receptors was not altered. In our previous study, we demonstrated that acute injection of BMS309043 had beneficial effects in alleviating constipation-like symptoms by accelerating colonic transit [22]. In the present study, the treatment with BMS309403 improved colonic propulsion and promoted forward movement of the contents of the small intestine toward the colon, thereby relieving constipation but only in the group supplemented with MCFA (COCO + BMS). Whether these effects occurred due to changes in the expression of µ opioid receptors still needs to be verified.

The active principle of castor oil is known to change the electrolyte permeability of the intestinal membrane and through elevated prostaglandin biosynthesis and release it causes diarrhea—similar to pathophysiologic conditions that cause diarrhea in humans [60]. In a castor oil-induced diarrhea, the supplementation with MCFAs (COCO group) as well as with LCFAs (EP group) had preventive effects against diarrhea by prolonging the time to appearance of liquid faeces. MCFAs and LCFAs could induce such changes by delaying transit in either upper or lower GI tract, or by increasing absorption of water and electrolytes through the intestinal wall, which consequently slowed down the secretion of fluid in the GI tract. Chronic treatment with BMS309403 significantly accelerated GI transit, when compared to dietary intervention without BMS309403 administration. It indicates that BMS309403 antagonizes the effect of diets in the model of castor oil-induced diarrhea by inhibiting the transport of FAs through the intestinal wall in FABP4-dependent manner.

In patients with FGIDs, modulatory mechanisms that regulate the sensory-reflex pathways are abnormal, and depending on the region affected, can lead to symptom generation or exacerbation. Several studies showed that intraduodenal infusion of fat (Intralipid solution) can affect the responses of the gut to different stimuli leading to gut hypersensitivity and disturbed reflexes [61–63]. However, it is worth mentioning that the major ingredient of the Intralipid solution is soybean oil, which in turn is a source of PUFAs, particularly FA composed of 18 carbon atoms. In our study, EPO increased pain perception at the highest pressure, which indicates that animals were more sensitive to CRD than animals from STD or COCO groups. A study incorporating a 28-day-long dietary intervention showed that rats fed with 3.5% of coconut oil or evening primrose oil inhibited colonic sensitivity to mechanical stimulation at the lower pressure values (15 and 30 mmHg), but had no influence on abdominal muscle motor function (measured as the duration of contractions) [57]. At higher pressure values (60 and 75 mmHg), rats exhibited similar sensitivity to mechanical stimulation, including the number, duration and the % of time with abdominal contraction to a group fed with 3.5% of soybean oil [57]. The effects of evening primrose oil on pain perception varied between studies. The differences may stem from the time of feeding, the concentration of FAs in the diet and the type of stimulation used to elicit contractions, i.e., phasic stimulation in the previous study on rats, and tonic stimulation in the current study.

The administration of BMS309403 reversed the effects caused by EPO. If the observed changes in visceral sensation were dependent on the type of FAs in a diet, it is possible that the effects were mediated through FABP4 activation since it has higher affinity towards the LCFAs.

In our earlier studies, we examined the presence and distribution of FABP4 and its possible co-expression with neuronal and endothelial markers in the colon, and verified whether BMS309403 can elicit functional changes in neuronal input to the circular smooth muscle in vivo [22]. We demonstrated that FABP4 was not co-localized with neuronal marker—beta tubulin, and did not induce electrophysiological changes in membrane potential of isolated colonic muscles. Likewise, in the present study, BMS309403 did not alter the sensation in response to CRD. Thus, taking all the findings together, we can rule out the hypothesis of the possible interaction of FABP4 and neuronal afferent nerves in the mouse GI tract and exclude the involvement of BMS30403 in promoting visceral sensitivity or generating changes in colonic neurotransmission.

Supplementation with FAs can effectively regulate the body fat by increasing the volume of adipocytes; therefore, in our studies, we also focused on analysis of primary fat cells, and examined the effects of diets and BMS309403 on preadipocyte proliferation and differentiation. We showed that proliferation of preadipocytes was not affected by the supplementation with COCO; however, it was significantly hampered by the treatment with EPO. The observed effect of EPO on preadipocyte proliferation is in accordance with other studies, which documented the anti-proliferative effects of LCFAs (particularly conjugated LA) on preadipocytes, which impeded the growth of lipid depots [64]. The treatment with BMS309403 substantially decreased the proliferation of COCO + BMS-derived cells and increased the proliferation in the EPO + BMS group.

FABP4 is highly expressed in mature adipocytes. Studies showed that expression of *Fabp4* is regulated mostly by saturated and monounsaturated FAs. However, more recently, it has been proven that LCFAs inhibit adipocyte differentiation and lipid accumulation in vitro, contributing to a decrease in the expression and consecutive long-term secretion, but not short-term secretion, of *Fabp4* [65, 66]. In the present study, COCO diet did not influence adipogenesis at the 1st and 3rd days since initiation of differentiation but it significantly reduced adipogenesis at the last day of differentiation, by decreasing the expression of *Fabp4*. In vitro studies on 3T3-L1 primary cells demonstrated that treatment with coconut oil, CLA or with lauric acid reduced fat accumulation in adipocyte during their differentiation, and decreased the expression of adipogenic nuclear factors [67]. Moreover, they failed to increase the level of *Fabp4* mRNA after induction of differentiation. In contrast, cells from the EPO group displayed the highest expression of *Fabp4* at the 1st day following the induction of differentiation and significantly lowered its expression at 6th day, when compared to STD group.

Although dietary supplementation with MCFAs and LCFAs significantly decreased adipogenesis at 6th day (since initiation of differentiation), our results suggest that adipogenesis process depends on the uptake of circulating FAs. The differentiation of preadipocytes at day 6 was higher in EPO group than COCO group indicating that supplementation with LCFA contributed to higher uptake of FAs, and thus higher lipid accumulation. This result can be related to high concentration of LA in EPO group. Generally, LA is thought to depress preadipocytes proliferation and de novo lipogenesis, and reduce fat accumulation in growing animals [68]. LCFAs activate a metabolic switch and contribute to lipid catabolism and suppression of lipogenesis, which also explains decrease in body weight of animals from the EPO group. However, it has to be emphasized that depending on the content of LA in a diet, LCFAs can exert different effects on adipogenesis. Even though STD diet contained LAs, the concentration of this FA was lower than in EPO diet, and was associated with increased differentiation of preadipocytes at 6th day. In line, treatment with stearidonic acid suppressed adipocyte differentiation and lipid accumulation by reducing the expression of transcription factors, including FABP4 [69]. Therefore, qualitative but not quantitative change in LCFAs can reduce *Fabp4* expression [65], and so, various sources of LCFAs can exert different effects on its expression. Moreover, as suggested by Prostak et al. [66], the influence of LCFAs can be age related, i.e., young cells seem to be more sensitive to LCFAs than mature and old ones.

BMS309403 appears to inhibit differentiation of pre-adipocyte at an early stage of differentiation in both COCO + BMS and EPO + BMS groups. However, it exerted the highest inhibitory potency towards COCO-derived mature adipocytes, and not towards differentiated mature EPO cells. BMS309403 can differently influence adipogenesis and the *Fabp4* gene expression, which may be related to the chain length or degree of unsaturation of FAs.

The present study is subject to some limitations. First, we did not incorporate animal models of IBS induced by alternating exposure to stress, e.g., maternal separation or the water avoidance stress, which in light of our observations could provide clearer results regarding the role of dietary interventions on the GI motility. Second, the consistency of fecal pellets and the FA content in the fecal matter have not been evaluated. Third, we did not verify whether the changes in the mRNA expression of FABP4 are accompanied by changes in the protein expression (e.g., by performing the Western Blot or Immunohistochemistry).

## Conclusions

The results obtained herein are of significance for several reasons. First, our data imply that FA supplementation with MCFAs or LCFAs regulates GI motility and that the effect might be dependent on the presence of FABP4 in the intestines. Secondly, FAs affect GI transit differently in physiological conditions and in mouse models mimicking IBS symptoms, i.e., models of constipation and diarrhea. Moreover, administration of FABP4 inhibitor, BMS309403, triggers a shift in the effects of MCFAs on GI motility but it does not significantly change the pain perception in vivo. The control of the mRNA expression of FABP4 appears to play a dominant role in overall GI motility (in these settings). It remains to be established whether BMS309403 impacts the function of FABP4 that is present in the intestines, or inhibits FABP4 in adipocytes where it affects the adipocyte-derived signals that modulate GI motility. Finally, our findings highlight the importance of dietary FAs in maintaining body homeostasis and control the course of the disease, and suggest possible therapeutic use of a synthetic FABP4 inhibitor in GI disorders, particularly IBS.

## Electronic supplementary material

Below is the link to the electronic supplementary material.

Figure S-1. Representative electromyogram recordings to 60 mmHg pressure in mice exposed to different dietary interventions (STD, COCO or EPO diet) with or without the administration of BMS309403. Contractions were elicited by insufflating the balloon with 0.6 mL distilled water (which corresponds to the 60 mmHg pressure). The first 10 s represents the baseline period. The time of the distention (10 s) is denoted by the horizontal red line below each recording (TIFF 2142 kb)

## Abbreviations

CLA: Conjugated LA
COCO: Coconut oil
Ct: Threshold cycle
DMSO: Dimethyl sulfoxide
EPO: Evening primrose oil
FAs: Fatty acids
FABP4: Fatty acid binding protein 4
FGIDs: Functional gastrointestinal disorders
FODMAP: Fermentable oligo-, di-, monosaccharides and polyols
FPO: Fecal pellet output
i.p.: Intraperitoneal
KRBH: Krebs–Ringer HEPES buffer
IBS: Irritable bowel syndrome
IBS-A: Alternating IBS
IBS-C: Constipation-predominant IBS
IBS-D: Diarrhea-predominant IBS
LA: Linoleic acid
LCFAs: Long-chain fatty acids
MCFAs: Medium-chain fatty acids
PUFAs: Polyunsaturated fatty acids
RT: Room temperature
STD: Standard laboratory diet
VMR: Visceromotor response
WAT: White adipose tissue
