# Supplementary figures and images for: Chain length of dietary fatty acids determines gastrointestinal motility and visceromotor function in mice in a fatty acid binding protein 4-dependent manner
Source: Eur J Nutr. 2019 Sep 27;59(6):2481–96. doi: 10.1007/s00394-019-02094-2 (PMC7413912; doi:10.1007/s00394-019-02094-2)

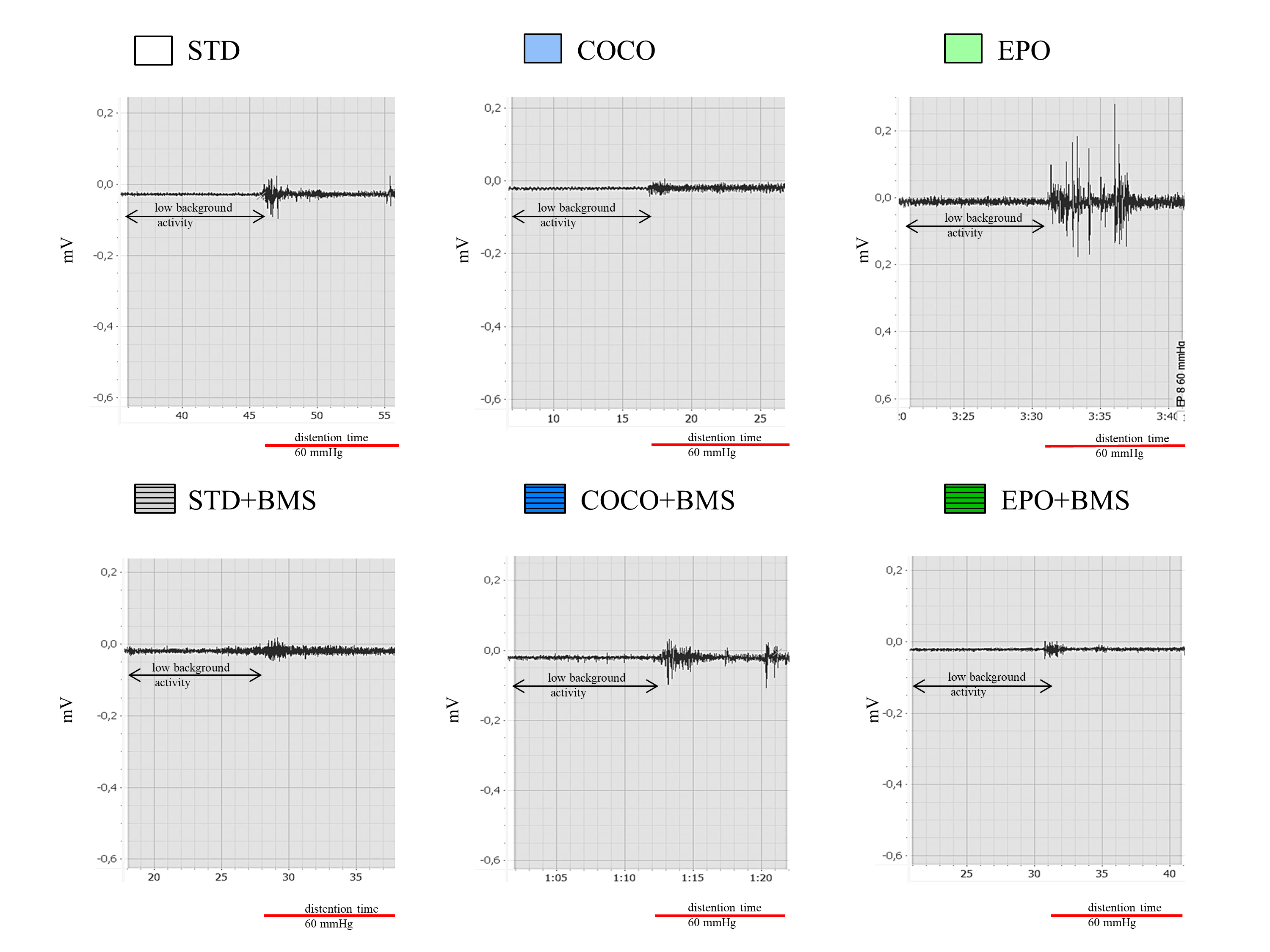

Supplement: Supplementary file 1 — Figure S-1. Representative electromyogram recordings to 60 mmHg pressure in mice exposed to different dietary interventions (STD, COCO or EPO diet) with or without the administration of BMS309403. Contractions were elicited by insufflating the balloon with 0.6 mL distilled water (which corresponds to the 60 mmHg pressure). The first 10 s represents the baseline period. The time of the distention (10 s) is denoted by the horizontal red line below each recording (TIFF 2142 kb) [file 394_2019_2094_MOESM1_ESM.tif]
